# Cell-Cycle Perturbations Suppress the Slow-Growth Defect of *spt10Δ* Mutants in *Saccharomyces cerevisiae*

**DOI:** 10.1534/g3.112.005389

**Published:** 2013-03-01

**Authors:** Jennifer S. Chang, Fred Winston

**Affiliations:** Department of Genetics, Harvard Medical School, Boston, Massachusetts 02115

**Keywords:** Spt10, Spt21, histones, suppressors

## Abstract

Spt10 is a putative acetyltransferase of *Saccharomyces cerevisiae* that directly activates the transcription of histone genes. Deletion of *SPT10* causes a severe slow growth phenotype, showing that Spt10 is critical for normal cell division. To gain insight into the function of Spt10, we identified mutations that impair or improve the growth of *spt10* null *(spt10Δ)* mutants. Mutations that cause lethality in combination with *spt10Δ* include particular components of the SAGA complex as well as *asf1Δ* and *hir1Δ*. Partial suppressors of the *spt10Δ* growth defect include mutations that perturb cell-cycle progression through the G1/S transition, S phase, and G2/M. Consistent with these results, slowing of cell-cycle progression by treatment with hydroxyurea or growth on medium containing glycerol as the carbon source also partially suppresses the *spt10Δ* slow-growth defect. In addition, mutations that impair the Lsm1-7−Pat1 complex, which regulates decapping of polyadenylated mRNAs, also partially suppress the *spt10Δ* growth defect. Interestingly, suppression of the *spt10Δ* growth defect is not accompanied by a restoration of normal histone mRNA levels. These findings suggest that Spt10 has multiple roles during cell division.

The *Saccharomyces cerevisiae* Spt10 protein plays important roles in gene expression and growth. Mutations in the *SPT10* gene have been identified in many different ways, including as suppressors of the transcriptional defects caused by Ty and Ty LTR insertion mutations (Fassler and Winston 1988; Natsoulis *et al.* 1991), suppressors of glucose repression of *ADH2* (Denis and Malvar 1990), and suppressors of loss of an upstream activation sequence (Prelich and Winston 1993; Yamashita 1993). Several subsequent studies have demonstrated that Spt10 is a site-specific DNA binding protein that binds cooperatively at the regulatory regions of the four *S. cerevisiae* histone loci where it activates transcription (Dollard *et al.* 1994; Eriksson *et al.* 2005, 2011; Hess *et al.* 2004; Mendiratta *et al.* 2006, 2007; Xu *et al.* 2005). DNA binding is dependent upon both a zinc finger domain and an adjacent region required for cooperative binding (Mendiratta *et al.* 2006, 2007). Spt10 also plays a negative role in histone gene transcription, as it is required for repression of several histone loci outside of S phase (Sherwood and Osley 1991). An intriguing feature of the Spt10 amino acid sequence is a conserved acetyltransferase domain (Neuwald and Landsman 1997). Although this domain is required for Spt10 function (Hess *et al.* 2004), no acetyltransferase activity or acetyltransferase substrates have yet been identified for Spt10, despite efforts by several laboratories.

The *SPT21* gene is functionally related to *SPT10*. Mutations in *SPT21* were isolated in two of the same mutant selections as mutations in *SPT10* (Natsoulis *et al.* 1991; Prelich and Winston 1993), including one large-scale selection that identified only these two genes (Natsoulis *et al.* 1991). In addition, mutations in *SPT21* appear to cause the same pattern of histone locus transcription defects as do mutations in *SPT10* (Dollard *et al.* 1994; Hess *et al.* 2004; Sherwood and Osley 1991). *In vivo*, Spt21 is also recruited to all four histone loci, and this recruitment is required for the recruitment of Spt10 during S-phase (Hess *et al.* 2004). Mutations in *SPT10* and *SPT21* share other phenotypes, including silencing defects (Chang and Winston 2011). Mutations have been identified in *SPT10* that suppress the requirement for *SPT21*, suggesting that Spt21 is an accessory factor, required for optimal Spt10 function (Hess *et al.* 2004).

In addition to the close functional relationships between *SPT10* and *SPT21*, obvious differences between them suggest that they do not always function together. There are three especially striking differences between the two. First, *SPT10* is transcribed throughout the cell cycle, whereas *SPT21* is transcribed only during S phase, at the same time as histone genes (Cho *et al.* 1998; Spellman *et al.* 1998). Second, a complete deletion of *SPT10 (spt10Δ)* causes a severe growth defect, whereas a complete deletion of *SPT21 (spt21Δ)* causes a only a mild growth defect (Natsoulis *et al.* 1994). Finally, mutations that suppress an *spt21Δ* mutation do not suppress *spt10Δ* and, in fact, sometimes cause lethality when combined with *spt10Δ* (Hess and Winston 2005). Taken together, the common and distinct phenotypes of *spt10Δ* and *spt21Δ* mutants suggest that Spt10 and Spt21 function together to regulate histone gene expression and that, in addition, Spt10 plays other roles that are critical for normal growth.

To gain insight into other possible roles for Spt10, we have screened for both enhancers and suppressors of the *spt10Δ* growth defect. The identification of mutations that cause lethality when combined with *spt10Δ* suggests that Spt10 has overlapping roles with the SAGA coactivator complex. In addition, Spt10 appears to be functionally related to Asf1, the Hir complex, and the Caf-1 complex, whose functions are connected in histone gene regulation, transcriptional silencing, and chromatin assembly (Amin *et al.* 2012; Eriksson *et al.* 2012; Kaufman *et al.* 1998; Sutton *et al.* 2001). The identification of partial suppressors of the *spt10Δ* growth defect suggests that Spt10 plays important roles throughout the cell cycle. In support of the idea that these functions are independent of the role of Spt10 as an activator of histone gene transcription, suppressors of the *spt10Δ* growth defect do not reverse the defects in histone gene transcription.

## Materials and Methods

### Yeast strains, media, and crosses

All *S. cerevisiae* strains (Table 1) are *GAL2^+^* derivatives of the S288C background (Winston *et al.* 1995). Capital letters denote wild-type genes, lowercase letters denote mutant alleles, and *Δ* indicates a complete open reading frame deletion. To construct *spt10Δ* haploids, the open reading frame of *SPT10* was first replaced with the *LEU2* gene or a kanamycin resistance marker in a diploid strain. Then, plasmid pFW217 (*SPT10-URA3-CEN)* was used to transform the diploid to Ura^+^, followed by sporulation of the diploid to obtain haploids with the *spt10Δ* mutation and pFW217. Whenever possible, *spt10Δ* strains were grown in the presence of pFW217 to minimize selection for spontaneous growth suppressors. Then, the *spt10Δ* phenotypes were tested after growth on medium with 5-fluoroorotic acid (5-FOA) to select for cells that had lost pFW217. For the *nap1Δ*::*kanMX*, *hsl1Δ*::*kanMX*, *mih1Δ*::*kanMX*, *swe1Δ*::*kanMX*, and *pat1Δ*::*kanMX* alleles, a 2.4-kb cassette was amplified by polymerase chain reaction (PCR) from genomic DNA isolated from the corresponding deletion set strain (Giaever *et al.* 2002), then used to transform a wild-type strain. The cassette contains a replacement of the entire open reading frame with a kanamycin resistance marker. The *cln3Δ*::*HIS3*, *lsm1Δ*::*natMX*, and *bck2Δ*::*hphMX* alleles were generated by PCR-mediated disruption of the entire open reading frame (Goldstein and McCusker 1999). All deletions were confirmed by PCR. The *cdc28-T18A Y19F* allele was generated by digesting p433 (a generous gift from A. Amon) with *Eco*RI and using the fragment containing the *cdc28-T18A Y19F* allele and the *URA3* marker to transform a wild-type strain. The *URA3* gene was then replaced with the *KanMX* drug resistance cassette of pRS400. Media, basic yeast techniques, mating, sporulation, and tetrad dissection were as previously described (Rose *et al.* 1990). Crosses to test double mutant lethality generally contained one parent with an *spt10Δ* mutation and also carrying plasmid pFW217 *(SPT10-URA3-CEN)*. Double-mutant lethality was assayed by replica plating the spore colonies to 5-FOA plates to determine whether strains that had lost pFW217 were viable.

**Table 1:** *S. cerevisiae* strains used in this study

Name	Genotype
FY2191	*MAT***a** *spt10Δ201*::*HIS3 lys2-128δ ura3-52 his3Δ200 leu2Δ1* + pFW217 (*SPT10-URA3-CEN*)
FY2915	*MAT***a** *hsl7-gs65f*::*Tn3-LEU2 spt10Δ201*::*HIS3 lys2-128δ ura3-52 his3Δ200 leu2Δ1*
FY2916	*MAT***a** *hsl7-gs63f*::*Tn3-LEU2 spt10Δ201*::*HIS3 lys2-128δ ura3-52 his3Δ200 leu2Δ1*
FY2917	*MAT***a** *lsm1-68f*::*Tn3-LEU2 spt10Δ201*::*HIS3 lys2-128δ ura3-52 his3Δ200 leu2Δ1*
FY2918	*MAT***a** *asf1-69c*::*Tn3-LEU2 spt10Δ201*::*HIS3 lys2-128δ ura3-52 his3Δ200 leu2Δ1*
FY2919	*MAT***a** *asf1-57b*::*Tn3-LEU2 spt10Δ201*::*HIS3 lys2-128δ ura3-52 his3Δ200 leu2Δ1*
FY2920	*MAT***a** *ydr333c-710a*::*Tn3-LEU2 spt10Δ201*::*HIS3 lys2-128δ ura3-52 his3Δ200 leu2Δ1*
FY2921	*MAT***a** *dbf2-719a*::*Tn3-LEU2 spt10Δ201*::*HIS3 lys2-128δ ura3-52 his3Δ200 leu2Δ1*
FY2922	*MAT***a** *lea1-719d*::*Tn3-LEU2 spt10Δ201*::*HIS3 lys2-128δ ura3-52 his3Δ200 leu2Δ1*
FY2923	*MATα spt10Δ*::*LEU2 can1Δ*::*STE2pr-HIS3 lys2-128d ura3Δ0 his3Δ1 or Δ200 leu2Δ0 lyp1Δ or LYP1* + pFW217 *(SPT10-URA3-CEN)*
FY2200	*MAT***a** *lys2-128δ ura3Δ0 his3Δ200 leu2Δ0*
FY2924	*MAT***a** *spt10Δ*::*LEU2 lys2-128δ ura3Δ0 his3Δ200 leu2Δ0* + pFW217 *(SPT10-URA3-CEN)*
FY2925	*MAT***a** *spt8-302*::*LEU2 spt10Δ*::*kanMX lys2-128δ* or *LYS2-173R2 ura3-52 leu2Δ1 trp1Δ63* + pFW217 *(SPT10-URA3-CEN)*
FY2926	*MAT***a** *spt20Δ200*::*ARG4 spt10Δ*::*LEU2 lys2-128δ* or *LYS2-173R2 ura3Δ0* or *-52 leu2Δ0* + pFW217 *(SPT10-URA3-CEN)*
FY2927	*MAT*α* gcn5Δ*::*HIS3 spt10Δ*::*LEU2 ura3Δ0 or ura3-52 his3Δ200 leu2Δ0 or leu2Δ1 his3Δ200 + pFW217 (SPT10-URA3-CEN)*
FY2928	*MAT***a** *ubp8Δ*::*kanMX4 spt10Δ*::*LEU2 lys2-128δ* or *LYS2-173R2 ura3Δ0* or *-52 his3Δ200 leu2Δ0* or *leu2Δ1 arg4-12* + pFW217 *(SPT10-URA3-CEN)*
FY2482	*MAT*α *spt21Δ*::*kanMX lys2-128δ ura3Δ0 his3Δ200 leu2Δ0*
FY2929	*MAT***a** *(hta2-htb2)Δ*::*URA3 hhf2Δ*::*LEU2 ura3-52 his3Δ200 leu2Δ1*
FY2930	*MAT***a** *hsl7Δ*::*HIS3 spt10Δ*::*LEU2 lys2-128δ ura3Δ0 his3Δ200 leu2Δ0* + pFW217 *(SPT10-URA3-CEN)*
FY2931	*MAT***a** *nap1Δ*::*kanMX spt10Δ*::*LEU2 lys2-128δ ura3Δ0 his3Δ200 leu2Δ0* + pFW217 *(SPT10-URA3-CEN)*
FY2932	*MAT***a** *bck2Δ*::*hphMX spt10Δ*::*LEU2 lys2-128δ ura3Δ0 his3Δ200 leu2Δ0* + pFW217 *(SPT10-URA3-CEN)*
FY2933	*MAT***a** *lsm1Δ*::*natMX spt10Δ*::*LEU2 lys2-128δ ura3Δ0 his3Δ200 leu2Δ0* + pFW217 *(SPT10-URA3-CEN)*
FY2934	*MAT***a** *hsl7Δ*::*HIS3 ura3Δ0 his3Δ200 leu2Δ0*
FY2935	*MAT***a** *nap1Δ*::*kanMX lys2-128δ ura3Δ0 his3Δ200 leu2Δ0*
FY2936	*MAT***a** *bck2Δ*::*hphMX lys2-128δ ura3Δ0 his3Δ200 leu2Δ0*
FY2937	*MAT***a** *lsm1Δ*::*natMX lys2-128δ ura3Δ0 his3Δ200 leu2Δ0*
FY2938	*MATα spt10Δ*::*LEU2 lys2-128δ ura3Δ0 his3Δ200 leu2Δ0* + pFW217 *(SPT10-URA3-CEN)*
FY2939	*MAT*a *hsl7Δ*::*HIS3 nap1Δ*::*kanMX spt10Δ*::*LEU2 ura3Δ0 his3Δ200 leu2Δ0* + pFW217 *(SPT10-URA3-CEN)*
FY2940	*MAT***a** *hsl7Δ*::*HIS3 bck2Δ*::*hphMX spt10Δ*::*LEU2 lys2-128δ ura3Δ0 his3Δ200 leu2Δ0* + pFW217 *(SPT10-URA3-CEN)*
FY2941	*MAT***a** *hsl7Δ*::*HIS3 lsm1Δ*::*natMX spt10Δ*::*LEU2 lys2-128δ ura3Δ0 his3Δ200 leu2Δ0*
FY2942	*MAT***a** *nap1Δ*::*kanMX bck2Δ*::*hphMX spt10Δ*::*LEU2 lys2-128δ ura3Δ0 his3Δ200 leu2Δ0* + pFW217 *(SPT10-URA3-CEN)*
FY2943	*MAT***a** *nap1Δ*::*kanMX lsm1Δ*::nat*MX spt10Δ*::*LEU2 lys2-128δ ura3Δ0 his3Δ200 leu2Δ0* + pFW217 *(SPT10-URA3-CEN)*
FY2944	*MAT***a** *bck2Δ*::*hphMX lsm1Δ*::*natMX spt10Δ*::*LEU2 lys2-128δ ura3Δ0 his3Δ200 leu2Δ0*
FY2945	*MAT***a** *hsl7Δ*::*HIS3 nap1Δ*::*kanMX bck2Δ*::*hphMX spt10Δ*::*LEU2 lys2-128δ ura3Δ0 his3Δ200 leu2Δ0* + pFW217 *(SPT10-URA3-CEN)*
FY2946	*MAT***a** *hsl7Δ*::*HIS3 nap1Δ*::*kanMX lsm1Δ*::*natMX spt10Δ*::*LEU2 lys2-128δ ura3Δ0 his3Δ200 leu2Δ0*
FY2947	*MAT***a** *hsl7Δ*::*HIS3 bck2Δ*::*hphMX lsm1Δ*::*natMX spt10Δ*::*LEU2 lys2-128δ ura3Δ0 his3Δ200 leu2Δ0*
FY2948	*MAT***a** *nap1Δ*::*kanMX bck2Δ*::*hphMX lsm1Δ*::*natMX spt10Δ*::*LEU2 lys2-128δ ura3Δ0 his3Δ200 leu2Δ0*
FY2949	*MAT***a** *hsl7Δ*::*HIS3 nap1Δ*::*kanMX bck2Δ*::*hphMX lsm1Δ*::*natMX spt10Δ*::*LEU2 lys2-128δ ura3Δ0 his3Δ200 leu2Δ0*
FY1856	*MAT*α *lys2-128δ ura3Δ0 his3Δ200 leu2Δ0*
FY2950	*MATα hsl7Δ*::*HIS3 spt10Δ*::*LEU2 ura3Δ0 his3Δ200 leu2Δ0* + pFW217 *(SPT10-URA3-CEN)*
FY2951	*MAT***a** *hsl1Δ*::*kanMX4 spt10Δ*::*LEU2 lys2-128δ ura3Δ0 his3Δ200 leu2Δ0* + pFW217 *(SPT10-URA3-CEN)*
FY2952	*MAT***a** *mih1Δ*::*kanMX4 spt10Δ*::*LEU2 lys2-128δ ura3Δ0 his3Δ200 leu2Δ0* + pFW217 *(SPT10-URA3-CEN)*
FY2953	*MAT***a** *swe1Δ*::*kanMX4 spt10Δ*::*LEU2 lys2-128δ ura3Δ0 his3Δ200 leu2Δ0* + pFW217 *(SPT10-URA3-CEN)*
FY2954	*MAT***a** *hsl7Δ*::*HIS3 swe1Δ*::*kanMX4 spt10Δ*::*LEU2 ura3Δ0 his3Δ200 leu2Δ0* + pFW217 *(SPT10-URA3-CEN)*
FY2955	*MAT***a** *hsl1Δ*::*kanMX4 lys2-128δ ura3Δ0 his3Δ200 leu2Δ0*
FY2956	*MAT***a** *mih1Δ*::*kanMX4 lys2-128δ ura3Δ0 his3Δ200 leu2Δ0*
FY2957	*MAT***a** *swe1Δ*::*kanMX4 lys2-128δ ura3Δ0 his3Δ200 leu2Δ0*
FY2958	*MAT***a** *cdc28-T18A Y19F:kanMX lys2-128δ ura3Δ0 his3Δ200 leu2Δ0*
FY2959	*MAT***a** *cdc28-T18A Y19F:kanMX spt10Δ*::*LEU2 lys2-128δ ura3Δ0 his3Δ200 leu2Δ0* + pFW217 *(SPT10-URA3-CEN)*
FY2960	*MAT***a** *hsl7Δ*::*HIS3 cdc28-T18A Y19F:kanMX spt10Δ*::*LEU2 ura3Δ0 his3Δ200 leu2Δ0* + pFW217 *(SPT10-URA3-CEN)*
FY2961	*MAT***a** *hsl7Δ*::*HIS3 cdc28-T18A Y19F:kanMX lys2-128δ ura3Δ0 his3Δ200 leu2Δ0*
FY2962	*MAT***a** *cln3Δ*::*HIS3 lys2-128δ ura3Δ0 his3Δ200 leu2Δ0*
FY2963	*MAT***a** *cln3Δ*::*HIS3 spt10Δ*::*LEU2 lys2-128δ ura3Δ0 his3Δ200 leu2Δ0* + pFW217 *(SPT10-URA3-CEN)*
FY2964	*MAT***a** *pat1Δ*::*kanMX spt10Δ*::*LEU2 lys2-128δ ura3Δ0 his3Δ200 leu2Δ0* + pFW217 *(SPT10-URA3-CEN)*
FY2965	*MAT***a** *pat1Δ*::*kanMX lsm1Δ*::*natMX spt10Δ*::*LEU2 lys2-128δ ura3Δ0 his3Δ200 leu2Δ0* + pFW217 *(SPT10-URA3-CEN)*
FY2966	*MAT***a** *pat1Δ*::*kanMX4 lys2-128δ ura3Δ0 his3Δ200 leu2Δ0*
FY2967	*MAT***a** *mec1Δ*::*LEU2 sml1Δ*::*HIS3 lys2-128δ ura3Δ0 his3Δ200 leu2Δ0*
FY2816	*MAT***a** *spt21Δ*::*HIS3 lys2-128δ ura3Δ0 his3Δ200 leu2Δ0*
FY2817	*MAT*α *spt21Δ*::*HIS3 lys2-128δ ura3Δ0 his3Δ200 leu2Δ0*
FY2968	*MAT*α *nap1Δ*::*kanMX spt10Δ*::*LEU2 lys2-128δ ura3Δ0 his3Δ200 leu2Δ0* + pFW217 *(SPT10-URA3-CEN)*
FY2969	*MATα bck2Δ*::*hphMX spt10Δ*::*LEU2 lys2-128δ ura3Δ0 his3Δ200 leu2Δ0* + pFW217 *(SPT10-URA3-CEN)*
FY2970	*MATα lsm1Δ*::*natMX spt10Δ*::*LEU2 lys2-128δ ura3Δ0 his3Δ200 leu2Δ0* + pFW217 *(SPT10-URA3-CEN)*
FY2971	*MATα hsl7Δ*::*HIS3 lsm1Δ*::*natMX spt10Δ*::*LEU2 lys2-128δ ura3Δ0 his3Δ200 leu2Δ0*
FY2972	*MATα hsl7Δ*::*HIS3 bck2Δ*::*hphMX lsm1Δ*::*natMX spt10Δ*::*LEU2 lys2-128δ ura3Δ0 his3Δ200 leu2Δ0*
FY1924	*MAT*α *hsl7Δ*::*HIS3 ura3Δ0 his3Δ200 leu2Δ0 trp1Δ63*
FY2973	*MATα nap1Δ*::*kanMX lys2-128δ ura3Δ0 his3Δ200 leu2Δ0*
FY2974	*MATα bck2Δ*::*hphMX lys2-128δ ura3Δ0 his3Δ200 leu2Δ0*
FY2975	*MATα lsm1Δ*::*natMX lys2-128δ ura3Δ0 his3D200 leu2Δ0*
FY2978	*MAT***a** *spt10Δ*::*KanMX leu2Δ1 ura3-52 lys2-128δ his3Δ200 +* pFW217 *(SPT10-URA3-CEN)*
FY2979	*MATα asf1Δ*::*HIS3 leu2Δ0 ura3Δ0 lys2-128δ his3Δ200*
FY2980	*MAT***a** *hir1Δ*::*LEU2 his4-912δ HIS3 ura3Δ0 or ura3-52 lys2-128d leu2Δ0 or leu2Δ1*
FY2981	*MAT***a** *spt21Δ*::*HIS3 ura3Δ0 leu2Δ0 lys2-128δ his3Δ200*
FY2982	*MATα asf1Δ*::*HIS3 ura3Δ0 leu2Δ0 lys2-128δ his3Δ200*
FY2903	*MAT***a** *cac1Δ*::*KanMX leu2Δ0 ura3Δ0 lys2-128δ his3Δ200*
FY2933	*MATα spt21Δ*::*HIS3 ura3Δ0 leu2Δ0 lys2-128δ his3Δ200*
FY1235	*MATα hir1Δ*::*LEU2 leu2Δ1 ura3-52 lys2-128δ his4-912δ trp1Δ63*

### Transposon mutagenesis screen

The transposon mutagenesis screen was performed as described (Burns *et al.* 1994). In summary, the *LEU2*-marked library DNA was digested with *Not*I, then used to transform strain FY2191. Transformant colonies were selected on SC-Leu-Ura medium then replica plated to 5-FOA medium to select for cells that had lost pFW217 (*SPT10-URA3)*, leaving colonies containing the library insertion in an *spt10Δ* genetic background. Colonies that failed to grow were designated synthetic lethal candidates, and colonies growing more quickly than FY2191 were designated growth suppressor candidates. All candidates were purified to single colonies, which were then individually patched on SC-Leu medium followed by replica plating to verify the growth phenotype. All candidates remaining after this rescreening were purified and tested a third time. Each candidate was then crossed to an *spt10Δ leu2* strain to test whether the mutant phenotype cosegregated with the *LEU2* marker on the transposon. For the confirmed mutants, genomic DNA was isolated, and vectorette PCR was used to identify the location of each transposon insertion (Arnold and Hodgson 1991). As one growth suppressor candidate was tightly linked to the *SPT10* locus, instead of vectorette PCR, we used a candidate gene approach and by a combination of PCR and sequencing, demonstrated the insertion to be within *LSM1*.

### Synthetic genetic array (SGA) screen

A collection of yeast strains containing deletions of every nonessential gene was screened for phenotypes in an *spt10Δ* background using an SGA screen (Tong *et al.* 2001). The collection was spotted onto YPD plates with deletion set strains *hoΔ*::*KanMX*, *lys2Δ*::*KanMX*, and *lys12Δ*::*KanMX* spotted separately at the top and bottom of each plate as controls that do not affect *spt10Δ* growth. The array was mated by replica plating to a lawn with an *spt10Δ* strain (FY2923) containing a *can1*::*STE2pr-HIS3* allele and carrying the pFW217 (*SPT10-URA3*) plasmid. Diploids were selected on SC-Leu-Ura and sporulated on solid 1% potassium acetate medium supplemented with histidine, uracil, leucine, and lysine. *MAT***a** haploids that contain the deletion set mutation, *spt10Δ*, and the *SPT10* plasmid were selected by replica plating onto SC-Arg-His-Leu-Ura+canavanine+G418 medium. The cells were then replica plated to SC + 5-FOA medium to leave the mutant *spt10Δ* as the only *SPT10* allele present. Strains with better or worse growth compared with the control strains were identified and retested, and then tetrads were dissected to assay for 2:2 segregation and cosegregation of the suppression phenotype with the kanamycin resistance marker.

### Dilution spot tests

**Figure 1:**
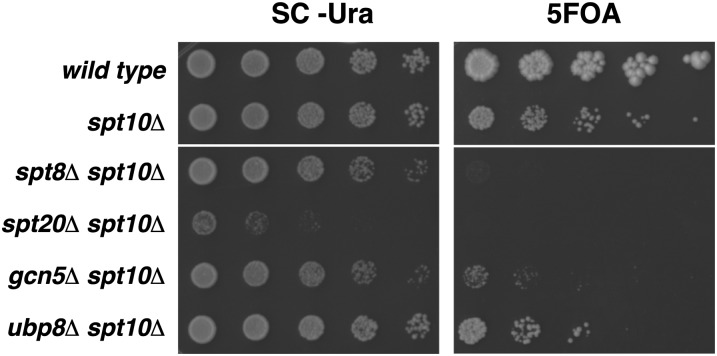
Mutations in genes encoding SAGA subunits lead to lethality or poor growth in an *spt10Δ* background. Shown are fivefold dilution spot tests. All strains were grown to saturation in SC-Ura medium in the presence of the pFW217 *SPT10-URA3-CEN* plasmid. They were serially diluted fivefold and spotted onto SC-Ura and 5-FOA plates to select for cells that have maintained or lost the *SPT10* plasmid, respectively. The SC-Ura plate is shown after 2 d of incubation at 30° and the 5-FOA plate after 5 d. Upper and lower panels are from the same plate. The strains were wild type (FY2200), *spt10Δ* (FY2924), *spt8Δ spt10Δ* (FY2925) *spt20Δ spt10Δ* (FY2926), *gcn5Δ spt10Δ* (FY2927), and *ubp8Δ spt10Δ* (FY2928).

**Figure 2:**
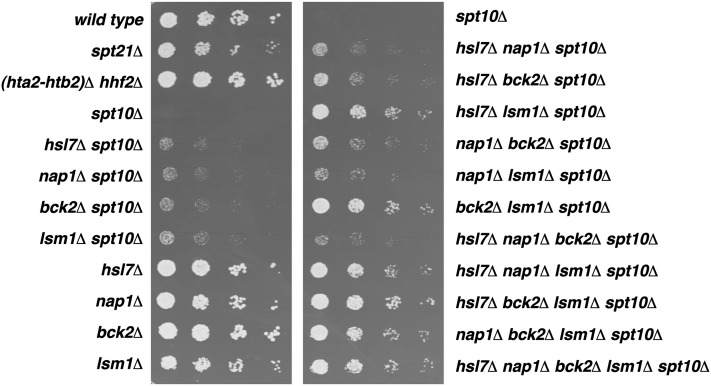
Representative suppressors of the *spt10Δ* slow growth phenotype. Shown are fivefold dilution spot tests. *spt10Δ* strains were cured of the pFW217 *SPT10-URA3-CEN* plasmid and grown as described in *Materials and Methods*, then resuspended to 4 × 10^6^ cells/mL. They were subjected to fivefold dilutions, spotted onto YPD medium, and photographed after 2 d. Strains were wild type (FY2200), *spt21Δ* (FY2482), *(hta2-htb2)Δ hhf2Δ* (FY2929), *spt10Δ* (FY2924), *hsl7Δ spt10Δ* (FY2930), *nap1Δ spt10Δ* (FY2931), *bck2Δ spt10Δ* (FY2932), *lsm1Δ spt10Δ* (FY2933), *hsl7Δ* (FY2934), *nap1Δ* (FY2935), *bck2Δ* (FY2936), *lsm1Δ* (FY2937), *spt10Δ* (FY2938), *hsl7Δ nap1Δ spt10Δ* (FY2939), *hsl7Δ bck2Δ spt10Δ* (FY2940), *hsl7Δ lsm1Δ spt10Δ* (FY2941), *nap1Δ bck2Δ spt10Δ* (FY2942), *nap1Δ lsm1Δ spt10Δ* (FY2943), *bck2Δ lsm1Δ spt10Δ* (FY2944), *hsl7Δ nap1Δ bck2Δ spt10Δ* (FY2945), *hsl7Δ nap1Δ lsm1Δ spt10Δ* (FY2946), *hsl7Δ bck2Δ lsm1Δ spt10Δ* (FY2947), *nap1Δ bck2Δ lsm1Δ spt10Δ* (FY2948), and *hsl7Δ nap1Δ bck2Δ lsm1Δ spt10Δ* (FY2949).

**Figure 3:**
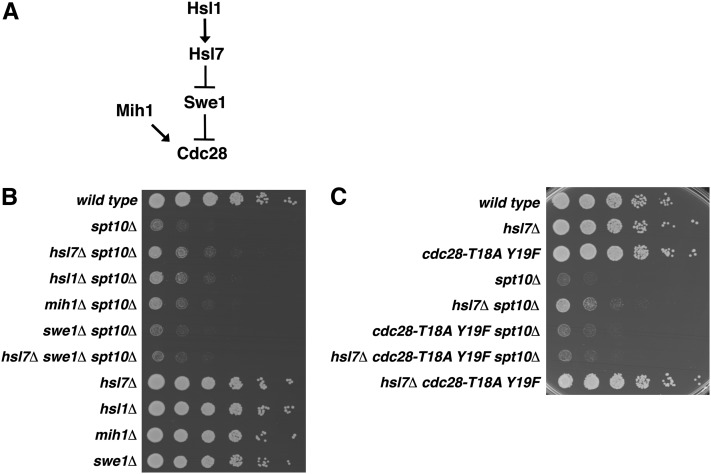
Perturbed progression through the bud morphogenesis checkpoint can suppress the *spt10Δ* growth defect. (A) Diagram of the Hsl−Swe1−Cdc28 pathway. (B, C) Fivefold dilution spot tests. Each strain was grown to saturation and diluted to 1.0 × 10^7^ cells/mL for the densest spot. Strains in (B) were wild type (FY2200), *spt10Δ* (FY2924), *hsl7Δ spt10Δ* (FY2930), *hsl1Δ spt10Δ* (FY2951), *mih1Δ spt10Δ* (FY2952), *swe1Δ spt10Δ* (FY2953), *hsl7Δ swe1Δ spt10Δ* (FY2954), *hsl7Δ* (FY2934), *hsl1Δ* (FY2955), *mih1Δ* (FY2956), and *swe1Δ* (FY2957). Strains in (C) were wild type (FY2200), *hsl7Δ* (FY2934), *cdc28-T18A Y19F* (FY2958), *spt10Δ* (FY2924), *hsl7Δ spt10Δ* (FY2930), *cdc28-T18A Y19F spt10Δ* (FY2959), *hsl7Δ cdc28-T18A Y19F spt10Δ* (FY2960), and *hsl7Δ cdc28-T18A Y19F* (FY2961). Pictures were taken after 2 d.

**Figure 4:**
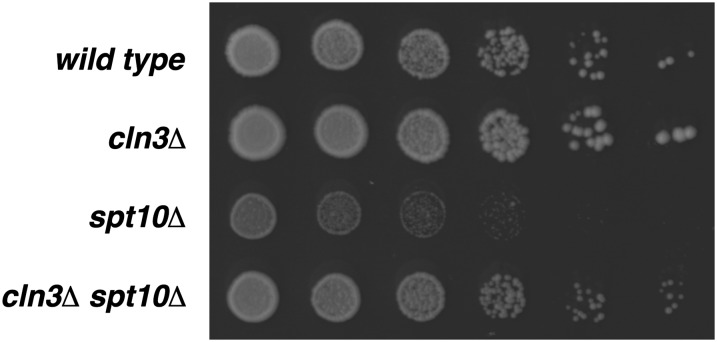
A mutation perturbing the G1/S transition can partially suppress the *spt10Δ* growth defect. Fivefold dilution spot assays were performed as in Figure 3. Strains were wild type (FY2200), *cln3Δ* (FY2962), *spt10Δ* (FY2924), and *cln3Δ spt10Δ* (FY2963). Pictures were taken after 2 d.

**Figure 5:**
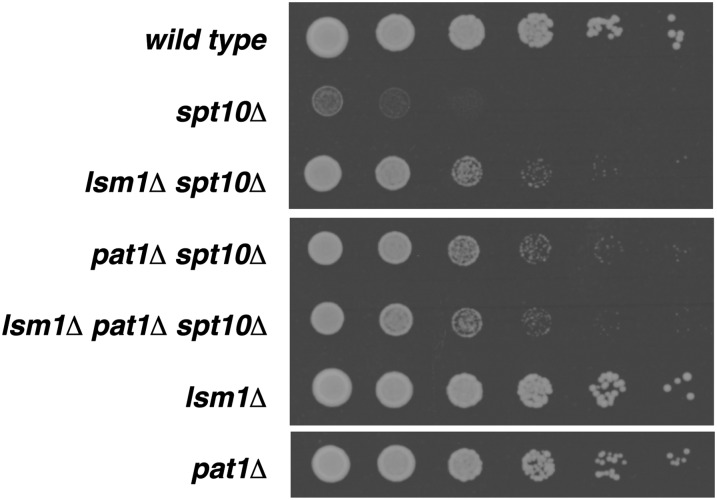
Suppression of the *spt10Δ* growth defect by mutations in the Lsm1-7-Pat1 complex. Dilution spot assays were performed as in Figure 3 with the following strains: wild type (FY2200), *spt10Δ* (FY2924), *lsm1Δ spt10Δ* (FY2933), *pat1Δ spt10Δ* (FY2964), *lsm1Δ pat1Δ spt10Δ* (FY2965), *lsm1Δ* (FY2937), and *pat1Δ* (FY2966). Pictures were taken after 2 d.

**Figure 6:**
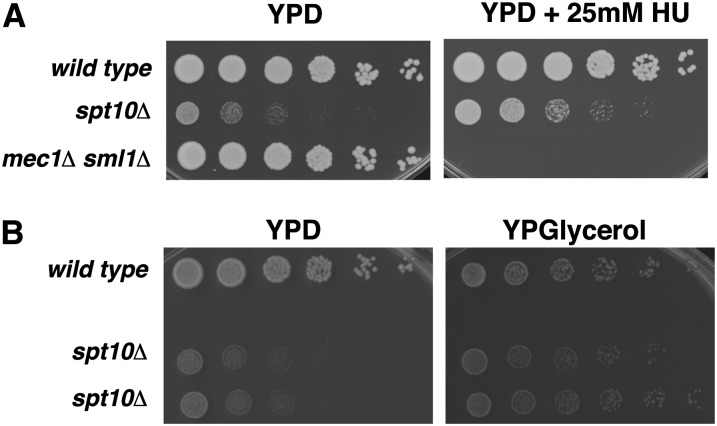
Nongenetic means of suppressing the *spt10Δ* slow growth phenotype. (A) Fivefold dilutions were made as in Figure 3, then spotted onto YPD medium or YPD + 25 mM HU. Pictures were taken after 2 d. Strains were WT (FY2200), *spt10Δ* (FY2924), and *mec1Δ sml1Δ* (FY2967). *mec1Δ sml1Δ* mutants are hypersensitive to HU. (B) Wild-type (FY2200) and *spt10∆* (FY2924) strains were subjected to fivefold serial dilutions as in Figure 3 and grown on YPD medium for two days or on YP + 3% glycerol medium for 5 d.

For dilution spot tests, unless noted otherwise, strains harboring the pFW217 *(SPT10-URA3-CEN)* plasmid were single colony purified on 5-FOA medium to select for plasmid loss, and single colonies were then patched to YPD media. After 2 d, the cells were resuspended in water to a density of 4 × 10^6^ cells/mL (Figure 2) or 1 × 10^7^ cells/mL (Figures 1, 3−6). Fivefold serial dilutions were spotted onto the media indicated. Plates were scanned after 2−3 d at 30°, unless otherwise indicated.

### cDNA synthesis and real-time PCR

RNA was extracted from 10 mL of yeast cultures in exponential growth as described (Ausubel *et al.* 1988; Swanson *et al.* 1991). Then, 10 μg of RNA was treated with 2 units of DNase (TURBO DNA free kit, Ambion) and reverse transcribed with Superscript III reverse transcriptase (Invitrogen) using an oligo-dT primer. Real-time PCR was performed with a Stratagene MX3000P machine using 50 ng of cDNA and 1 μg of each primer per 50 μL of reaction, with each reaction performed in triplicate. Primer sequences (Table 2) were provided by Neil McLaughlin and David Clark (personal communication). The specificity of each primer pair was confirmed using the corresponding deletion mutant. Thermocycling parameters were: 10:00 at 94°, then 35−40 cycles of (0:30 at 94°, 0:30 at 52°, 1:00 at 72°), followed by a melting curve to assay product specificity. Linearity and efficiency was confirmed for each primer pair on each plate.

**Table 2:** Primers used to measure histone mRNA levels

Primer	Gene	Orientation	Sequence
FO6006	*HTA1*	Forward	TTCAAAACAAACAAATTTCA
FO6007	*HTA1*	Reverse	AAATACCAGAACCGATCTTA
FO6008	*HTA2*	Forward	GGAAAGTACAGAACAAGAGC
FO6009	*HTA2*	Reverse	CTTTGTTTCTTTTCAACTCAG
FO6010	*HTB1*	Forward	CAAACCACAAATAAACCATAC
FO6011	*HTB1*	Reverse	AGGAAGTGATTTCATTATGC
FO6012	*HTB2*	Forward	ACCAACAACAACTTACTCTACA
FO6013	*HTB2*	Reverse	AATCACAATACCTAGTGAGTGA
FO6014	*HHT1*	Forward	TATATAAACGCAAACAATGG
FO6015	*HHT1*	Reverse	AACTGATGACAATCAACAAA
FO6016	*HHT2*	Forward	TACTAAAGGATCCAAGCAAA
FO6017	*HHT2*	Reverse	AAAAATTCCCGCTTTATATT
FO6018	*HHF1*	Forward	AACAAACAAAAACAAGCAAC
FO6019	*HHF1*	Reverse	TTGTTGTTACCGTTTTCTTA
FO6020	*HHF2*	Forward	GTAGCAAAAACAACAATCAA
FO6021	*HHF2*	Reverse	ATAATTTCAAACACCGATTG
FO6145	*ACT1*	Forward	TTTTGTCCTTGTACTCTTCC
FO6146	*ACT1*	Reverse	CTGAATCTTTCGTTACCAAT

## Results

### Identification of mutations that enhance or suppress the *spt10Δ* slow-growth phenotype

To study the basis of the *spt10Δ* slow growth phenotype, we screened for mutations that enhance or suppress the growth defect by using both transposon insertion mutagenesis (Burns *et al.* 1994) and the *S. cerevisiae* deletion set (Giaever *et al.* 2002), both as described in *Materials and Methods*. As spontaneous suppressors of the *spt10Δ* slow growth phenotype arise at a high frequency, we maintained a low-copy *SPT10* plasmid (pFW217) in the *spt10Δ* strains until the final screening step for each method.

We began with a transposon insertion mutagenesis screen (Burns *et al.* 1994; Kumar and Snyder 2002) in which we tested 9000 independent transformants for improved or impaired growth compared with the *spt10Δ* parent (*Materials and Methods*). By this approach, we identified eight mutations in a total of six genes (Table 3). Three mutations that confer suppression of *spt10Δ* poor growth were in two genes and five mutations that cause lethality when combined with *spt10Δ* were identified in four genes. For all six genes, we tested a complete deletion of the identified gene and found the same suppression phenotype, suggesting that all of the insertion mutations cause null phenotypes. For all subsequent experiments, the deletion mutations were used.

**Table 3:** Genes identified by a transposon screen

Gene	Effect When Combined With *spt10Δ*	Insertion Location Relative to ATG	Description
*HSL7*	Improved growth	+1232	Arginine N-methyltransferase involved in regulation of Swe1 degradation
*HSL7*	Improved growth	+1654	Arginine N-methyltransferase involved in regulation of Swe1 degradation
*LSM1*	Improved growth	−191	Part of a complex involved in degradation of cytoplasmic mRNAs
*ASF1*	Lethality	+102	Histone chaperone
*ASF1*	Lethality	+283	Histone chaperone
*YDR333C*	Lethality	+530	Unknown function
*DBF2*	Lethality	+1475	Ser/Thr kinase; exit from mitosis
*LEA1*	Lethality	+361	Component of U2 snRNP

From this initial screen, a concern of bias arose, as we had obtained two different transposon insertions within *ASF1* without obtaining any insertions in other genes whose deletions were previously shown to be lethal in combination with *spt10Δ*. These genes include *HTA1*, *HTB1*, *HHF1*, *HIR1*, *ASF1*, *RKR1*, and *MBP1* (Braun *et al.* 2007; Fassler and Winston 1988; Hess 2004; Hess and Winston 2005; Sutton *et al.* 2001). Therefore, rather than saturate the transposon mutagenesis screen, which would require testing 30,000 transformants (Burns *et al.* 1994), we switched to the more systematic approach of screening the deletion set.

We screened the deletion set for mutations that either suppress or enhance the *spt10Δ* slow growth defect (*Materials and Methods*). Our screen yielded 44 mutations that cause lethality in combination with *spt10Δ* (Table 4) and 13 mutations that improve *spt10Δ* growth (Table 5). Interestingly, there was no overlap with the mutations identified from the transposon mutagenesis screen, although some functionally related genes were identified (*LSM* genes). The lack of overlap indicates that the deletion set screen had many false-negative results. There was also a class of 12 mutants that appeared to cause lethality during the original screen but showed little or no growth defect upon tetrad dissection (discussed in the section *Genes involved in silencing show mutant phenotypes in combination with spt10Δ*).

**Table 4:** Genes found by SGA analysis whose deletion causes double-mutant lethality or extreme sickness with *spt10Δ*

Gene	Description
*BCK1*	MAP KKK in the protein kinase C signaling pathway
*BUD20*	Protein involved in bud site selection
*CAC2*	Component of chromatin assembly complex CAF-I
*CTF19*	Component of the COMA complex
*CYS3*	Cysteine biosynthesis
*DOA1*	Ubiquitin-mediated protein degradation
*ELP2*	Component of the Elongator complex
*ELP4*	Component of the Elongator complex
*ELP6*	Component of the Elongator complex
*HHF1*	Histone H4
*HHT1*	Histone H3
*HIR2*	Component of the HIR complex
*HIR3*	Component of the HIR complex
*HIT1*	Function unknown
*HPC2*	Component of the HIR complex
*IES2*	Associates with the INO80 chromatin remodeling complex
*IXR1*	Binds DNA containing intrastrand cross-links formed by cisplatin
*MCM21*	Component of the COMA complex
*MDM20*	Component of the NatB N-terminal acetyltransferase
*MRPL38*	Mitochondrial ribosomal protein of the large component
*MSD1*	Mitochondrial aspartyl-tRNA synthetase
*NHX1*	Endosomal Na^+^/H+ exchanger
*PEP7*	Facilitates vesicle-mediated vacuolar protein sorting
*PGD1*	Component of the mediator complex
*REG1*	Negative regulation of glucose-repressible genes
*RMD8*	Cytosolic protein required for sporulation
*SAM37*	Component of the mitochondrial SAM complex
*SGF11*	Component of the SAGA complex
*SGF29*	Component of the SAGA complex
*SIN3*	Component of the Rpd3-Sin3 complex
*SLX8*	Component of the Slx5-Slx8 SUMO-targeted ubiquitin ligase complex
*SOD1*	Cytosolic copper-zinc superoxide dismutase
*SPT3*	Component of the SAGA complex
*SPT8*	Component of the SAGA complex
*SWC3*	Component of the SWR1 complex
*TAF14*	Component of TFIID, TFIIF, INO80, SWI/SNF, and NuA3 complexes
*THR1*	Threonine synthesis
*THR4*	Threonine synthase
*UMP1*	Chaperone required for maturation of the 20S proteasome
*VMA8*	Component of the peripheral membrane domain of the vacuolar H+-ATPase
*VMS1*	Protein degradation and quality control
*VPS54*	Component of the GARP complex
*YAF9*	Component of both the NuA4 histone H4 and SWR1 complexes
*YGL149W*	Dubious open reading frame, overlaps *INO80*

**Table 5:** Genes found by SGA analysis whose deletion suppresses the *spt10Δ* poor growth phenotype

Gene	Description
*BCK2*	Protein kinase C signaling pathway and the G1/S transition
*CLB2*	B-type cyclin involved in G2 to M progression
*HAL5*	Putative protein kinase
*HDA2*	Component of a class II histone deacetylase complex
*IES3*	Component of the INO80 complex
*ITR1*	Myo-inositol transporter
*LAS21*	Synthesis of the glycosylphosphatidylinositol (GPI) core structure
*LSM6*	Part of complexes involved in RNA processing, splicing, and decay
*LSM7*	Part of complexes involved in RNA processing, splicing, and decay
*NAP1*	Bud morphogenesis, microtubule dynamics, and transport of histones H2A and H2B
*SIF2*	Component of the Set3C complex
*SLM4*	Component of the EGO complex
*SYH1*	Protein of unknown function, influences nuclear pore distribution

### The loss of specific classes of SAGA genes is lethal in combination with *spt10Δ*

Our screens identified four genes encoding components of the SAGA coactivator complex whose deletion is lethal when combined with *spt10Δ*: *SPT3*, *SPT8*, *SGF11*, *and SGF29*. These four factors are believed to be involved in distinct activities of the multifunctional SAGA complex, as Spt3 and Spt8 modulate the recruitment of the TATA-binding protein (TBP) to promoters (Bhaumik and Green 2001, 2002; Dudley *et al.* 1999; Larschan and Winston 2001), Sgf11 is part of the DUB module of SAGA (Kohler *et al.* 2010; Samara *et al.* 2010), and Sgf29 has recently been shown to bind to H3K4me2/3, to be required for Gcn5-dependent histone acetylation *in vivo*, and to help recruit TBP to promoters (Bian *et al.* 2011; Shukla *et al.* 2012). To test whether the double-mutant lethality with *spt10Δ* is general for all SAGA deletion mutants or specific for certain classes, we tested deletions of *SPT20*, encoding a core component of SAGA, *UBP8*, encoding a histone deubiquitylase, and *GCN5*, encoding the histone acetyltransferase. Our results (Figure 1) show that the *spt20Δ spt10Δ* double mutant is inviable, whereas both the *ubp8Δ spt10Δ* and *gcn5Δ spt10Δ* double mutants are viable but grow poorly, even worse than the *spt10Δ* single mutant. Our genetic analysis, then, demonstrates that Spt10 shares essential or important roles with distinct functions of the SAGA coactivator complex. In light of the *spt10Δ-gcn5Δ* genetic interaction, we note that we did not see a genetic interaction between *spt10Δ* and *rtt109Δ* (*RTT109* encodes a histone acetyltransferase that has been implicated in histone gene transcription) (Fillingham *et al.* 2009).

### Double-mutant lethality of *spt10Δ* with *asf1Δ* and *hir/hpc2Δ* mutations suggests functional overlaps

Among the genes identified as causing double-mutant lethality with *spt10Δ* were *asf1Δ*, *hir2Δ*, *hir3Δ*, and *hpc2Δ*. Previous studies also showed that *spt10Δ asf1Δ* double mutants are inviable (Sutton *et al.* 2001). Asf1 has been shown to be a histone chaperone (Munakata *et al.* 2000), the Hir complex (comprised of Hir1-3 and Hpc2) has been implicated in chaperone and nucleosome assembly activities (Green *et al.* 2005; Prochasson *et al.* 2005), and both Asf1 and the Hir complex have been shown to regulate histone gene transcription (Osley and Lycan 1987; Sutton *et al.* 2001; Xu *et al.* 1992). Furthermore, these factors are believed to function both physically and genetically with each other and with the Caf-1 complex (Green *et al.* 2005; Kaufman *et al.* 1998; Liu *et al.* 2012; Sutton *et al.* 2001).

The isolation of *asf1Δ* and *hir/hpc2Δ* mutations as causing lethality when combined with *spt10Δ* suggests that Spt10 participates in this set of functions. To test this further, we crossed *spt10Δ* by *hir1Δ* and by *cac1/rlf2Δ* (*CAC1* encodes a component of the Caf-1 complex) to test for double mutant lethality. Our results (Table 6) show that *spt10Δ* causes inviability with *asf1Δ* and *hir/hpc* mutations, but not with *cac1Δ*. This pattern is reminiscent of earlier studies that showed that both *asf1Δ* and *hir/hpc* mutations cause double-mutant sickness with *cac* mutations, but not with each other (Kaufman *et al.* 1998; Sutton *et al.* 2001). We note that our screens did not identify mutations in *RTT106*, which encodes a histone chaperone that has been shown to regulate histone gene transcription by interactions with Asf1/Hir/Caf-1 (Fillingham *et al.* 2009; Huang *et al.* 2007; Kurat *et al.* 2011; Silva *et al.* 2012; Zunder and Rine 2012). Similarly, a screen for mutations that cause double-mutant lethality with *rtt106Δ* did not identify *spt10Δ* (Imbeault *et al.* 2008). In contrast to *spt10Δ*, an *spt21Δ* mutation allowed viability when combined with *hir1Δ* or *asf1Δ* (Table 6). Taken together, our results suggest that Spt10, but not Spt21, contributes to an essential function in collaboration with Asf1 and the Hir complex, likely either in histone gene activation or an aspect of chromatin assembly.

**Table 6:** *spt10Δ* is inviable with *hir1Δ* and *asf1Δ*

Double Mutant	Phenotype*^a^*
*spt10Δ hir1Δ*	Inviable*^b^*
*spt10Δ asf1Δ*	Inviable*^c^*
*spt10Δ cac1Δ*	Viable*^d^*
*spt21Δ hir1Δ*	Viable*^e^*
*spt21Δ asf1Δ*	Viable*^f^*
*spt21Δ cac1Δ*	Viable*^g^*

a The phenotype was determined by testing the ability of the double mutant to survive loss of plasmid pFW217 *(SPT10-URA3-CEN)* by assaying growth on 5FOA plates as described in *Materials and Methods*. The cross done for each combination is listed below.

b FY2978 × FY1235.

c FY2924 × FY2979.

d FY2903 × FY2938.

e FY2980 × FY2933.

f FY2981 × FY2982.

g FY2903 x FY2933.

### Genes involved in silencing show mutant phenotypes in combination with *spt10Δ*

One notable class of mutants appeared to show lethality in combination with *spt10Δ* during our systematic screen. However, upon retesting by tetrad dissection, viable double mutant spores were obtained at the expected frequency, without substantial growth defects. This class of mutants included *sir1Δ*, *ard1Δ*, and *pol32Δ*, all of which have roles in silencing (Pillus and Rine 1989; van Welsem *et al.* 2008; Whiteway *et al.* 1987). Others have reported a similar pattern of apparent lethality for *sir1Δ dot1Δ* and *pol32Δ dot1Δ* in another deletion set screen (van Welsem *et al.* 2008). They discovered that the pattern actually resulted from mating type silencing defects, which prevent growth when the SGA screening method is used. Our studies of Spt10 have demonstrated it to be required for silencing (Chang and Winston 2011).

### The slow growth of *spt10Δ* mutants can be suppressed through multiple genetic pathways

The mutations that we identified that suppress the *spt10Δ* growth defect fall into several functional categories. For the remainder of our analysis, we focused on the four mutations that individually caused the strongest suppression of the *spt10Δ* growth defect: *hsl7Δ*, *nap1Δ*, *bck2Δ*, and *lsm1Δ* (Figure 2). Hsl7 is an arginine methyltransferase with a role in the bud morphogenesis checkpoint (Lew 2000). Nap1 is a histone chaperone involved in the nuclear import of histones, and it regulates cell-cycle progression in G2/M (Zlatanova *et al.* 2007). Bck2 regulates the transition from G1 to S phase of the cell cycle (Epstein and Cross 1994; Lee *et al.* 1993), and Lsm1 is part of a heteroheptameric complex involved in RNA decapping and processing (Tharun 2009). Lsm1 has recently been shown to control histone mRNA stability (Herrero and Moreno 2011). All of the deletion mutations are partial suppressors individually, but when *lsm1Δ* is combined with *hsl7Δ* or *bck2Δ*, strong additive effects are seen (Figure 2). Little or no additivity is seen with other combinations. This finding suggests that *hsl7Δ* and *bck2Δ* suppress the *spt10Δ* growth defect through a different genetic pathway than does *lsm1Δ*. To study these effects, we conducted a more detailed genetic analysis of each suppressor.

### Perturbations of the G2/M transition allow *spt10Δ* mutants to grow faster

*HSL7*, along with *HSL1*, initially was isolated in a *h*istone *s*ynthetic *l*ethal screen, which identified genes that become essential when the tail of either histone H3 or histone H4 is deleted (Ma *et al.* 1996). Although the basis of this synthetic lethality remains unknown, Hsl1, a protein kinase, and Hsl7 have been shown to regulate the bud morphogenesis checkpoint through the Hsl−Swe1−Cdc28 pathway, which monitors whether cytoskeletal events have been properly completed prior to mitosis (Figure 3A) (Lew 2000). The cyclin-dependent kinase Cdc28 controls cell-cycle progression through the G2/M transition; its activity is inhibited by the kinase Swe1 and activated by the phosphatase Mih1. When an *S. cerevisiae* cell buds, Hsl1 recruits Hsl7 to the bud neck and phosphorylates both proteins. This recruits Swe1, leading to Swe1 degradation, causing decreased phosphorylation of Cdc28 and thereby promoting progression through G2/M. Thus, an *hsl7Δ* single mutant has increased Swe1 activity, resulting in decreased Cdc28 activity. We tested the effects of other mutations in the Hsl−Swe1−Cdc28 pathway on *spt10Δ* growth. Consistent with our findings for *hsl7Δ*, both *hsl1Δ* and *mih1Δ*, which also impair progression through the bud morphogenesis checkpoint, suppress the *spt10Δ* growth defect, whereas a mutation (*swe1Δ*) that promotes progression does not (Figure 3B). As additional evidence that impairment of G2/M progression suppresses the *spt10Δ* growth defect, we identified *clb2Δ* as a suppressor in our screen (Table 5).

To test whether suppression of the *spt10Δ* growth defect by *hsl7Δ* occurs within the Hsl−Swe1−Cdc28 pathway, we tested combinations of mutations in this pathway. First, we found that *swe1Δ* is epistatic to *hsl7Δ* with respect to suppression of the *spt10Δ* growth defect (Figure 3B), suggesting that suppression by *hsl7Δ* is mediated through Swe1 activity. Second, we tested whether the inhibitory phosphorylation of Cdc28 by Swe1 plays a role in *hsl7Δ* suppression of the *spt10Δ* growth defect. To do this, we used the *cdc28-T18A Y19F* allele (Amon *et al.* 1992; Sorger and Murray 1992), which makes cells insensitive to mutations upstream in the Hsl-Swe1-Cdc28 pathway, thus mimicking loss of Swe1. We found that *hsl7Δ* no longer suppresses the *spt10Δ* growth defect in the presence of the *cdc28-T18A Y19F* allele (Figure 3C), further supporting that *hsl1Δ*- and *hsl7Δ*-mediated suppression occurs through the Hsl−Swe1−Cdc28 pathway. Taken together, our genetic analysis suggests that mutations that activate the bud morphogenesis checkpoint can confer improved growth of *spt10Δ* cells.

### Perturbations at the G1/S transition also suppress the *spt10Δ* growth defect

Bck2 was originally isolated as a factor important in protein kinase C signaling, and it has been found to be important in controlling the G1/S transition of the cell cycle (Epstein and Cross 1994; Lee *et al.* 1993). A related protein involved in regulating the G1/S transition is Cln3, a cyclin that binds to Cdc28 to regulate the transition through START (Richardson *et al.* 1989). We asked whether a *cln3Δ* mutation can also suppress the *spt10Δ* growth defect. Spot tests demonstrate that *cln3Δ spt10Δ* mutants grow better than *spt10Δ* single mutants (Figure 4), suggesting that different perturbations in the G1/S transition can suppress the *spt10Δ* growth defect. Taken together with the *hsl7Δ* suppression data, our genetic analysis demonstrates that the *spt10Δ* slow growth can be suppressed by mutations that delay cell cycle progression at either the G1/S transition or the bud morphogenesis G2/M checkpoint.

### Impairment of the Lsm1-7−Pat1 complex suppresses the *spt10Δ* slow growth phenotype

Next we conducted a more detailed genetic analysis of three closely related suppressors: *lsm1Δ*, *lsm6Δ*, and *lsm7Δ*. The eight *S. cerevisiae LSM* (like Sm) genes form two distinct, ring-shaped, heteroeptameric complexes (Tharun 2009). The first complex, containing Lsm2-8, localizes to the nucleus and regulates pre-mRNA splicing. The second complex, containing Lsm1-7, is localized to the cytoplasm and regulates the decapping of polyadenylated mRNAs, in conjunction with Pat1 (*p*rotein *a*ssociated with *T*opoisomerase II). We note that in both larger eukaryotes (Tharun 2009) and in yeast (Herrero and Moreno 2011), the Lsm1-7−Pat1 complex has been implicated in promoting the degradation of histone mRNAs.

The result that *lsm1Δ* suppresses the *spt10Δ* slow growth phenotype suggests that it is the Lsm1-7−Pat1 complex, rather than the Lsm2−Lsm8 complex that is related to *spt10Δ* growth. We therefore also tested whether *pat1Δ* suppresses the *spt10Δ* growth phenotype. Our results (Figure 5) show that *pat1Δ* does suppress the *spt10Δ* growth defect and, furthermore, that suppression by *lsm1Δ* and *pat1Δ* is not additive, suggesting that *lsm1Δ* and *pat1Δ* suppress the *spt10Δ* growth defect through the same pathway. The other *LSM* genes in the complex are essential for viability and could not be tested.

### Environmental conditions that slow cell division also suppress the *spt10Δ* slow growth phenotype

Considering that genetic means of slowing cell-cycle progression can suppress the *spt10Δ* slow growth phenotype, we asked whether altered growth conditions that slow cell cycle progression will also suppress this phenotype. First, we assayed the growth of *spt10Δ* strains on medium containing 25 mM hydroxyurea (HU), a ribonucleotide reductase inhibitor that impedes S-phase progression. We found that addition of 25 mM HU causes modest suppression of the *spt10Δ* growth defect relative to wild-type growth (Figure 6A).

Second, we slowed growth using medium that contains glycerol rather than glucose as a carbon source. Relative to wild-type, *spt10Δ* growth modestly improves on this medium (Figure 6B). These findings are consistent with the possibility that slowing cell cycle progression through multiple means improves *spt10Δ* growth.

### Suppressors of the *spt10Δ* growth phenotype do not restore histone mRNA levels

Because Spt10 binds to histone gene promoters and regulates histone gene transcription (Dollard *et al.* 1994; Eriksson *et al.* 2011; Hess *et al.* 2004; Sherwood and Osley 1991; Xu *et al.* 2005), we wanted to test whether the suppressors improve *spt10Δ* growth by increasing histone gene mRNA levels. We therefore measured mRNA levels for all eight histone genes in the suppressor strains, using reverse transcription and real-time PCR. We used primer pairs highly specific for their corresponding transcripts (Table 2; N. McLaughlin and D. Clark, personal communication) to distinguish the two nearly identical copies of each histone gene.

**Figure 7:**
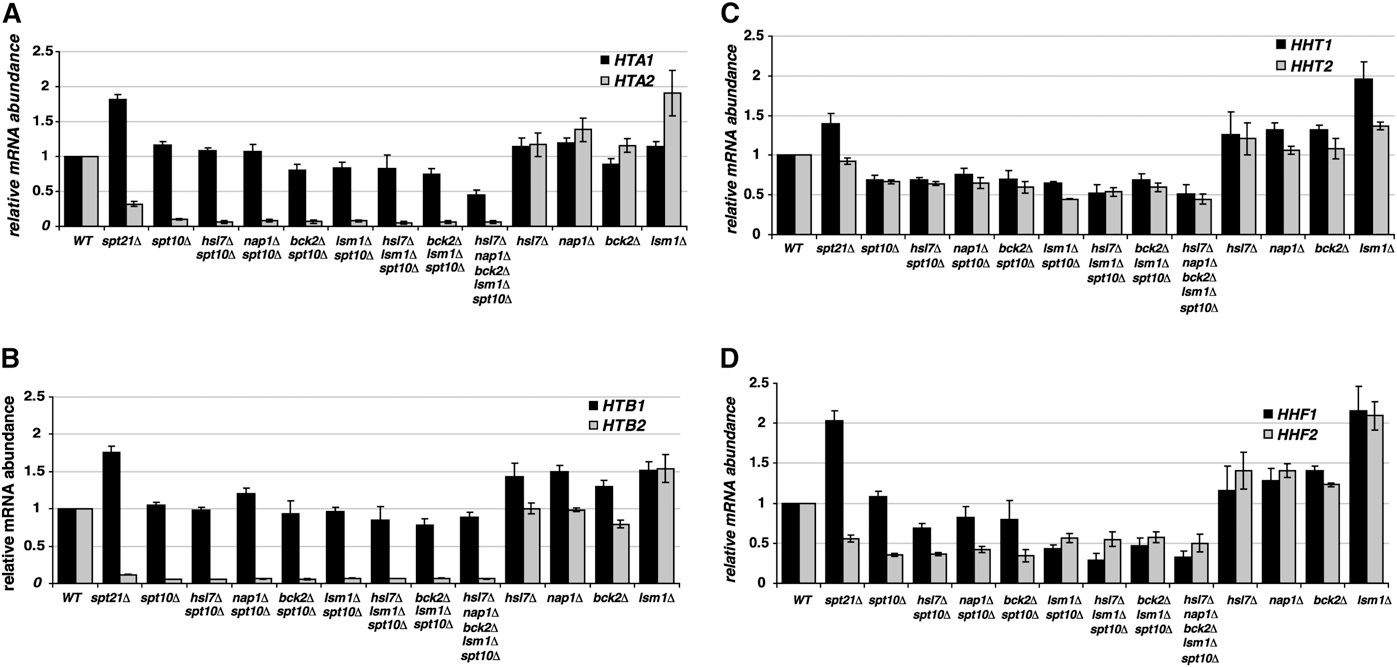
mRNA abundance for the core histone genes in growth suppressor strains. RNA was isolated and reverse transcribed, and real-time PCR with gene-specific primers (Table 2) was used to quantitate histone mRNA levels for (A) *HTA1* and *HTA2*; (B) *HTB1* and *HTB2*; (C) *HHT1* and *HHT2*; and (D) *HHF1* and *HHF2*. All values were normalized to *ACT1* mRNA levels and are shown relative to wild type, which was assigned a value of 1. Shown is the mean ± SEM for at least three independent experiments. Strains were wild type (FY2200 and FY1856), *spt10Δ* (FY2924 and FY2938), *spt21Δ* (FY2816 and FY2817), *hsl7Δ spt10Δ* (FY2930 and FY2950), *nap1Δ spt10Δ* (FY2931 and FY2968), *bck2Δ spt10Δ* (FY2932 and FY2969), *lsm1Δ spt10Δ* (FY2933 and FY2970), *hsl7Δ lsm1Δ spt10Δ* (FY2941 and FY2971), *bck2Δ lsm1Δ spt10Δ* (FY2944), *hsl7Δ nap1Δ bck2Δ lsm1Δ spt10Δ* (FY2949 and FY2972), *hsl7Δ* (FY2934 and FY1924), *nap1Δ* (FY2935, FY2973), *bck2Δ* (FY2936, FY2974), and *lsm1Δ* (FY2937, FY2975).

Our results (Figure 7) show that the suppressors do not restore histone mRNA levels in an *spt10Δ* background. First, in agreement with previous results (Dollard *et al.* 1994; Hess *et al.* 2004), we found that, in asynchronously growing cultures, *HTA2* and *HTB2* mRNA levels are decreased approximately 20-fold, with more modest decreases of *HHT1*, *HHT2*, and *HHF2* mRNA levels. In an *spt10Δ* background, no single suppressor mutation or multiple suppressor combination restores mRNA levels for any histone gene. The only substantial change with any suppressor mutation is a decrease in *HHF1* mRNA levels in *spt10Δ* mutants when *LSM1* is deleted. This is in spite of the finding that some of the suppressor mutations cause modest changes in histone mRNA levels in a wild-type *SPT10* background. The increased level of histone mRNAs observed for *lsm1Δ* agrees with previous results (Herrero and Moreno 2011). Overall, our results suggest that restoration of normal histone mRNA levels is not necessary for suppression of the *spt10Δ* slow growth phenotype.

We note that, like *spt10∆* mutants, *spt21∆* mutants show decreased levels of *HTA2*, *HTB2*, and *HHF2* mRNA, but unlike *spt10∆* mutants or the suppressor strains, the *spt21∆* mutants show modest increases in mRNA levels for *HTA1*, *HTB1*, *HHF1*, and to a lesser degree *HHT1*. These results suggest that Spt10 and Spt21 have some non-overlapping roles in histone gene regulation.

## Discussion

In this work, we have identified a broad spectrum of mutations that either cause lethality when combined with *spt10Δ* or that suppress the slow growth phenotype caused by *spt10Δ*. The first set of genes suggests that the function of Spt10 partially overlaps with the SAGA coactivator complex as well as with two factors involved in chromatin assembly and histone gene transcription, Asf1 and the Hir complex. Given the pleiotropic nature of mutants lacking these functions, as well as the documented role of Asf1 and the Hir complex in histone gene regulation (Osley and Lycan 1987; Sutton *et al.* 2001; Xu *et al.* 1992), these double mutant lethalities are not surprising. Several additional genes were identified in the screen for double-mutant lethality (Tables 3 and 4), and the results suggest that functional overlaps also exist between Spt10 and both the Elongator complex and the Ino80 complex. As there are no known roles for SAGA, Elongator, or Ino80 in histone gene expression, further studies of these interactions will be required to understand whether the essential process in which Spt10 and these other factors participate involves histone gene expression or a previously uncharacterized role for Spt10.

The suppressors of the *spt10Δ* growth defect led us to conclude that perturbations at multiple points of the cell cycle can suppress the slow growth of *spt10Δ* mutants. Although it seems paradoxical that an impairment of cell-cycle progression would enhance growth, there is precedent for a defect in one process suppressing a defect in a related process. For example, a cold-sensitive *spt5* mutation is suppressed with 6-azauracil, which decreases the rate of transcription elongation (Hartzog *et al.* 1998). Furthermore, perturbations in multiple different cell cycle phases can suppress a silencing defect at the *S. cerevisiae* silent mating type loci and telomeres (Laman *et al.* 1995).

One model to explain our findings is that *spt10Δ* mutants grow slowly due to the shortage of a factor or factors necessary for normal growth, and that cell cycle perturbations compensate for this growth-limitation, either by allowing more time for the factor to be produced, or by adjusting the relative levels of factors with which it interacts. Considering the well-characterized role of Spt10 in activating histone gene transcription, obvious candidates for such factors are histone proteins. We note that histone levels are clearly a factor in *spt10Δ* growth, as a plasmid that encodes all four core histones (with the *HTA1-HTB1* and *HHT1-HHF1* loci) restores *spt10Δ* growth to nearly wild-type levels (Eriksson *et al.* 2005; Silva *et al.* 2012). However, we found that suppressors of the *spt10Δ* growth defect do not suppress the *spt10Δ* defect in histone mRNA levels, suggesting that the slow growth can be affected by other routes, possibly independent of histone gene transcription. Alternatively, the suppressors might partially alleviate the requirement for normal histone levels.

Left unresolved by these and other studies of Spt10 is the role of the Spt10 acetyltransferase domain. While it is required for Spt10 function (Hess *et al.* 2004), its target(s) remain unknown. The elucidation of these targets will go a long ways toward helping us understand the roles of Spt10 in growth.
